# A Pre-mRNA–Associating Factor Links Endogenous siRNAs to Chromatin Regulation

**DOI:** 10.1371/journal.pgen.1002249

**Published:** 2011-08-25

**Authors:** Kirk B. Burkhart, Shouhong Guang, Bethany A. Buckley, Lily Wong, Aaron F. Bochner, Scott Kennedy

**Affiliations:** 1 Laboratory of Genetics, University of Wisconsin Madison, Madison, Wisconsin, United States of America; 2 Department of Pharmacology, University of Wisconsin Madison, Madison, Wisconsin, United States of America; Massachusetts General Hospital, Howard Hughes Medical Institute, United States of America

## Abstract

In plants and fungi, small RNAs silence gene expression in the nucleus by establishing repressive chromatin states. The role of endogenous small RNAs in metazoan nuclei is largely unknown. Here we show that endogenous small interfering RNAs (endo-siRNAs) direct Histone H3 Lysine 9 methylation (H3K9me) in *Caenorhabditis elegans*. In addition, we report the identification and characterization of *nuclear RNAi defective* (*nrde*)*-1* and *nrde-4*. Endo-siRNA–driven H3K9me requires the nuclear RNAi pathway including the Argonaute (Ago) NRDE-3, the conserved nuclear RNAi factor NRDE-2, as well as NRDE-1 and NRDE-4. Small RNAs direct NRDE-1 to associate with the pre-mRNA and chromatin of genes, which have been targeted by RNAi. NRDE-3 and NRDE-2 are required for the association of NRDE-1 with pre-mRNA and chromatin. NRDE-4 is required for NRDE-1/chromatin association, but not NRDE-1/pre-mRNA association. These data establish that NRDE-1 is a novel pre-mRNA and chromatin-associating factor that links small RNAs to H3K9 methylation. In addition, these results demonstrate that endo-siRNAs direct chromatin modifications via the Nrde pathway in *C. elegans*.

## Author Summary

Chromatin consists of DNA and proteins. Chromatin can exist in many different states. The state of chromatin in highly regulated in order to ensure that genes are expressed correctly. RNAs play an important role in the regulation of chromatin. For example, in plants and fungi small RNAs drive the formation of heterochromatin, a repressive chromatin state. Many types of small RNAs have been identified in animal cells, but the functions of these small RNAs are largely unknown. Using the nematode *C. elegans* as a model system, we identified a small RNA pathway that regulates the state of chromatin. We report the identification of two new factors, termed NRDE-1 and NRDE-4, which act in this nuclear small RNA pathway. NRDE-1 and NRDE-4 link small RNAs to chromatin regulation. Additionally, we show that endogenously expressed small RNAs, termed the endo-siRNAs, direct the post-translational modification of histone proteins, which are core components of chromatin. These results establish a direct connection between small RNAs and chromatin regulation in animals.

## Introduction

Small regulatory RNAs can silence gene expression in the nucleus by establishing repressive chromatin states. This process, termed Transcriptional Gene Silencing (TGS), was first observed in plants, where small RNAs direct DNA methylation and histone modifications (reviewed in [1]). In addition, the fission yeast, *Schizosaccharomyces pombe* has been an important model in defining the role of small RNAs in heterochromatin formation. In *S. pombe*, small RNAs direct the formation of heterochromatin primarily at repetitive DNA elements surrounding centromeres [2], [3]. At these repetitive elements, nascent RNAs, transcribed by RNA Polymerase II (RNAP II), serve as platforms for the assembly of RNAi machinery. For instance, the RNA Induced Transcriptional Silencing (RITS) complex, composed of the Argonaute Ago1, the chromodomain protein Chp1, and the glycine and tryptophan (GW)-motif-containing protein Tas3, is guided to nascent transcripts by Argonaute and centromeric siRNAs [4]. The RITS complex recruits chromatin-modifying machinery, such as the histone methyltransferase Clr4, to genomic sites of nuclear RNAi [5], [6]. Clr4 catalyzes the methylation of Histone H3 on Lysine 9 (H3K9me) [7]. H3K9me is a conserved molecular mark of heterochromatin [8]. Thus, in plants and *S. pombe*, small RNAs play a central role in regulating chromatin dynamics. The role of TGS and heterochromatin formation in metazoan silencing processes is less clear [3].

Experimentally provided small RNAs can elicit transcriptional silencing and induce heterochromatic marks in metazoans. In mammalian cells, experimentally provided siRNAs directed against promoter regions can lead to transcriptional silencing and induce heterochromatic marks [9]–[12]. Paradoxically, experimentally provided small RNAs can also enhance transcription and decrease H3K9me marks [13], [14]. In *C. elegans*, experimentally provided siRNAs are bound by the Ago NRDE-3 in the cytoplasm, and escorted into the nucleus [15]. NRDE-3/siRNA ribonucleoprotein complexes bind nascent transcripts and recruit the conserved nuclear RNAi factor NRDE-2. The Nrde pathway inhibits RNA Polymerase (RNAP) II during the elongation phase of transcription, and directs the deposition of H3K9me marks at genomic sites that exhibit homology to experimentally introduced siRNAs [16].

How and if endogenously expressed small regulatory RNAs silence gene expression in metazoan nuclei is unclear. Dicer deficient mouse embryonic stem cells express high levels of centromeric repeat RNAs and exhibit altered heterochromatic marks at centromeres [17]. In Drosophila, heterochromatic marks, including H3K9me and HP1, are mislocalized in flies lacking components of the RNAi machinery such as Piwi, Aubergine, and Homeless [18]. In addition, the Drosophila Ago-like protein PIWI binds small RNAs, termed piRNAs, and associates with chromatin [19]. Loss of *piwi* has variable effects on chromatin states at genomic sites homologous to piRNAs [20]–[24]. Finally, in *C. elegans*, animals lacking two RNAi-related factors: the RNA-dependent RNA Polymerase EGO-1, or the Ago CSR-1, exhibit large-scale changes in chromosomal H3K9me patterns during germline development [25], [26]. Thus, endogenous small regulatory RNAs have been implicated in chromatin regulation in metazoans. However, a direct link has yet to be established, and the molecular mechanisms by which this might occur are unknown.

Here we show that the endogenous small RNAs, termed endo-siRNAs, direct H3K9me marks at discrete genomic loci in *C. elegans*. Small RNA-directed H3K9 methylation requires the Nrde pathway and results in the inhibition of transcription from these loci. In addition, we identify two novel nuclear RNAi factors termed NRDE-1 and NRDE-4, and show that these factors are required for small RNA-directed H3K9 methylation. Finally, we show that small RNAs direct NRDE-1 to associate with pre-mRNA and chromatin of genes, which have been targeted by RNAi. Thus, the Nrde pathway links endogenously expressed small regulatory RNAs to the regulation of transcription and chromatin dynamics in *C. elegans*.

## Results

### A genetic screen identifies novel *nrde* genes

**Figure 1:**
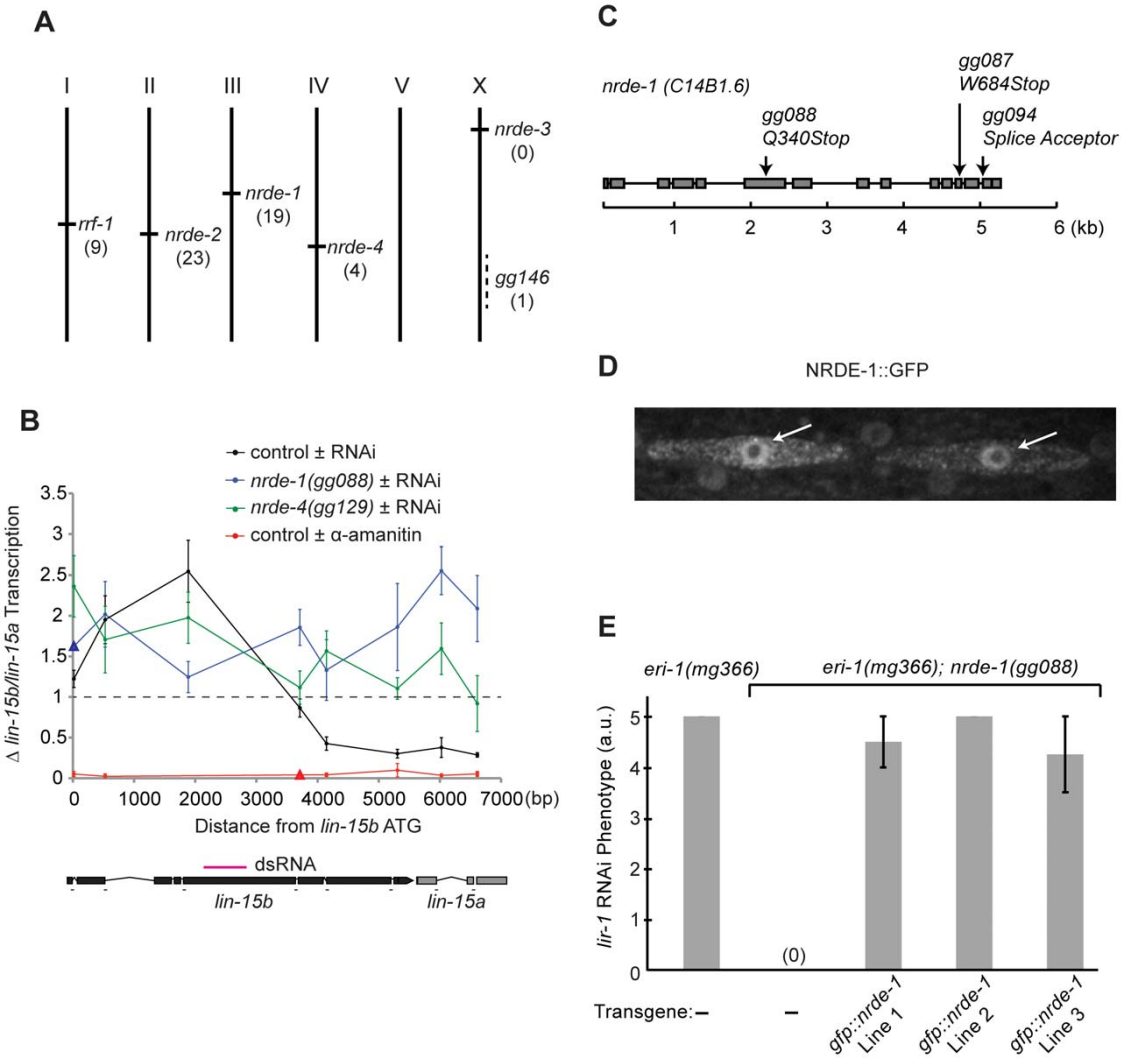
*nrde-1* encodes a nuclear-localized protein that is required for nuclear RNAi. (A) Genetic map positions of genes identified in genetic screen. Number of alleles identified in screen are indicated. (B) RNAi-driven transcriptional inhibition requires *nrde-1* and *nrde-4*. Nuclear Run On (NRO) analysis of transcription from the *lin-15b/a* gene from nuclei of animals exposed to +/− *lin-15b* dsRNA or +/− α-amanitin. Data are expressed as a ratio +/− *lin-15b* dsRNA (normalized to transcription detected from *eft-3* gene) or +/− α-amanitin (5 µg/ml). Dotted line indicates a ratio of 1; i.e. no change. The genetic background for this experiment was *eri-1(mg366)*. Control +/− *lin-15b* dsRNA (n = 3–8, +/− s.e.m.), *nrde-1(gg088)* (n = 4–6, +/− s.e.m., data point represented by triangle n = 1), *nrde-4(gg129)*(n = 5, +/− s.e.m.), control +/− α-amanitin (n = 3 +/− s.e.m., data point represented by triangle n = 1). Δ = fold change. Below: diagram of *lin-15b/lin-15a* gene structure indicating location of primers and trigger dsRNA (magenta). (C) Predicted *nrde-1* gene structure. Arrows indicate mutant alleles. (D) NRDE-1 localizes to the nucleus. Fluorescence microscopy of two seam cells in a L4 larval animal expressing *gfp::nrde-1*. Arrows indicate nuclei. (E) *gfp::nrde-1* fusion gene rescues *nrde-1* mutant phenotype. Animals of the indicated genotypes were exposed to *lir-1* dsRNA. A score of 5 indicates all animals died during larval development and a score of 0 indicates animals did not exhibit any developmental defects. Plates were scored blind and in triplicate for *lir-1* RNAi-mediated lethality (a.u. arbitrary units).

We previously reported a forward genetic screen that identified two genes (termed *nrde-2* and *nrde-3*) required for nuclear RNAi [15], [16]. The mechanism(s) by which NRDE-2/3 silence nascent transcripts and inhibit RNAP II transcription are unknown. To understand this mechanism we continued screening for nuclear RNAi factors. >80% of the *nrde* alleles identified in our original genetic screen were alleles of *nrde-3* (Table S1, [15]). To maximize our chances of identifying novel nuclear RNAi factors, we performed our modified screen in animals harboring ectopic copies of *nrde-3* (*nrde-3::gfp*), which was integrated into the genome on chromosome V (Figure S1). *eri-1* encodes an exonuclease that negatively regulates RNAi [27]. Our original screen was conducted in an *eri-1(−)* genetic background. Our modified screen was conducted in *eri-1(+)* animals (Figure S1). Our modified screen identified twenty-three alleles of *nrde-2*, nineteen alleles of *nrde-1*, nine alleles of the RNA-dependent RNA Polymerase (RdRP) *rrf-1*, four alleles of *nrde-4*, and one additional *nrde* allele, which complements the known *nrde* genes, but has not yet been assigned a *nrde* gene designation (Figure 1a, and Table S1). Here we report the identification and characterization of *nrde-1* and *nrde-4*.

### *nrde-1* is a component of the nuclear RNAi pathway

We first focused our attention on characterizing the role of *nrde-1* in nuclear RNAi. Three lines of evidence indicate that *nrde-1* functions with *nrde-2* and *nrde-3* to silence nuclear-localized RNAs during nuclear RNAi. First, NRDE-1, like NRDE-2/3, is required for RNAi-based silencing of nuclear-localized RNAs. For instance, the *lir-1* and *lin-26* genes are expressed in an operon; these genes are co-transcribed as a polycistronic pre-mRNA, which is spliced into distinct mRNAs in the nucleus before export to the cytoplasm [28], [29]. *lir-1(−)* mutant animals are viable, whereas *lin-26(−)* mutant animals exhibit a lethal phenotype [30]. RNAi targeting *lir-1* induces a lethal phenotype, indicating that *lir-1* RNAi silences the nuclear-localized *lir-1/lin-26* RNA [15], [30]. *nrde-2(−)* and *nrde-3(−)* animals are viable following *lir-1* RNAi, indicating that NRDE-2 and NRDE-3 are required for *lir-1*-mediated silencing of the *lir-1/lin-26* RNA [15], [16]. *nrde-1* mutant animals were also viable when exposed to *lir-1* RNAi, indicating that, like NRDE-2 and NRDE-3, NRDE-1 is required for silencing of the *lir-1/lin-26* RNA (Table 1). Similarly, NRDE-1/2/3 are required to silence the nuclear-localized *lin-15b/lin-15a* RNA. *lin-15b* and *lin-15a* genes are encoded in an operon. Mutations in *lin-15b* or *lin-15a* alone produce no obvious phenotype, but animals harboring mutations in both *lin-15b* and *lin-15a* exhibit a Multi-vulva (Muv) phenotype [31]. RNAi targeting *lin-15b* induces a Muv phenotype, indicating that *lin-15b* RNAi silences the nuclear-localized *lin-15b/lin-15a* RNA [15], [16]. *nrde-1/2/3* mutant animals do not exhibit a Muv phenotype in response to *lin-15b* RNAi, indicating that NRDE-1/2/3 are required for silencing the *lin-15b/lin-15a* RNA (Table 1). Second, nuclear-localized siRNAs direct a NRDE-2/3 dependent inhibition of RNAP II during the elongation phase of transcription [16]. For instance, *lin-15b* RNAi inhibits RNAP II transcription 3′ to the site of *lin-15b* RNAi (Figure 1b). RNAi-mediated inhibition of RNAP II transcription is dependent upon NRDE-2 and NRDE-3 [15], [16]. *nrde-1* was also required to link small RNAs to RNAP II inhibition; in *nrde-1* mutant animals, *lin-15b* RNAi did not result in transcription inhibition (Figure 1b). Thus, like *nrde-2/3*, a wild-type copy of the *nrde-*1 gene is required for RNAi to inhibit transcription elongation. Third, we conducted a genetic analysis using double mutant combinations of the Nrde factors. This analysis indicated that *nrde-1* functions in a genetic pathway with *nrde-2* and *nrde-3* (Figure S2). Taken together, these data argue that *nrde-1* is a component of the Nrde silencing pathway.

**Table 1:** *nrde-1* is required for silencing nuclear localized RNAs.

	dsRNA	
	(phenotype scored)	
	*lin-15b*	*lir-1*
Genotype	(Multi-vulva)	(Lethality)
*eri-1(mg366)*	+	+
*eri-1(mg366); rde-1(ne219)*	−	−
*eri-1(mg366); nrde-1(gg088)*	−	−
*eri-1(mg366); nrde-4(gg129)*	−	−
*eri-1(mg366); nrde-1(gg088); nrde-2(gg091)*	−	−
*eri-1(mg366); nrde-1(gg088); nrde-3(gg066)*	−	−
*eri-1(mg366); nrde-1(gg088); nrde-4(gg129)*	−	−

Animals of the indicated genotypes were fed bacteria expressing indicated dsRNAs (e.g. *lin-15b*). *eri-1* encodes an exonuclease that is required for the biogenesis of endogenous small interfering RNAs (endo-siRNAs) [36]. *eri-1(−)* animals have an enhanced RNAi phenotype; they respond more robustly to dsRNA than wild-type animals [27]. Therefore, an *eri-1(−)* background was used to facilitate phenotypic analysis. The phenotypes (e.g. Multi-vulva) of *eri-1(mg366)* animals exposed to dsRNA were defined as ‘+’ (∼90–100% of animals with phenotype), the phenotypes of *eri-1(mg366);rde-1(ne219)* were defined as ‘−’ (0% of animals with phenotype). 50–250 animals were scored blind in each trial (n≥3).

### NRDE-1 is a nuclear-localized protein

To determine the molecular identity of *nrde-1*, we used a single nucleotide polymorphism (SNP)-based mapping approach [32]. We mapped *nrde-1* to a 0.86cM interval on Chromosome III that contained 42 genes. The open reading frame (ORF) *c14b1.6* lies within this mapping interval. Sequencing of *c14b1.6* from three independent *nrde-1* alleles revealed three mutations in *c14b1.6* (Figure 1c). Two of these alleles encode premature stop codons, and therefore likely reveal the null phenotype of *nrde-1*. Expression of a wild-type copy of *c14b1.6* was sufficient to rescue the Nrde phenotype associated with *nrde-1* (see below). We conclude that *c14b1.6* corresponds to *nrde-1*. Analysis of *nrde-1* expressed sequence tags (ESTs) indicated that *nrde-1* encodes a protein containing 793 amino acids [33]. Database searches revealed that *nrde-1* is conserved in other nematode species, but these searches failed to detect any obvious orthologues of *nrde-1* outside nematodes. In addition, these database searches did not identify any obvious protein domains within NRDE-1.

We assessed the sub-cellular distribution of NRDE-1. We constructed a NRDE-1 and Green Fluorescent Protein fusion protein (NRDE-1::GFP), which encodes GFP 5′ to a full length copy of *nrde-1*. We observed fluorescence in nuclei of NRDE-1::GFP expressing animals (Figure 1d). NRDE-1::GFP rescued Nrde phenotypes associated with *nrde-1(−)* animals (Figure 1e), suggesting that the NRDE-1::GFP expression pattern reflects the expression pattern of endogenous NRDE-1. We conclude that NRDE-1 is a nuclear localized protein.

### NRDE-1 functions downstream of NRDE-3

**Figure 2:**
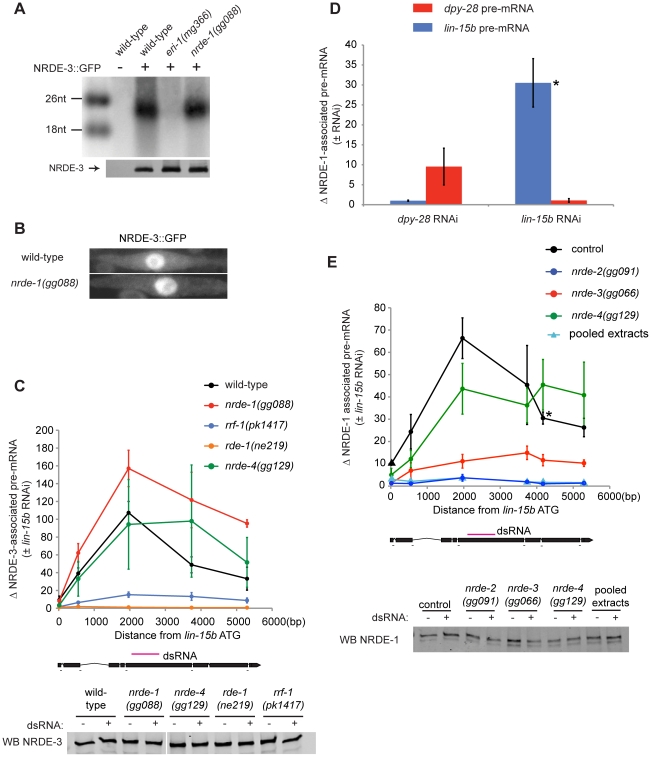
NRDE-1 is recruited by NRDE-2/-3 to pre-mRNAs that have been targeted by RNAi. (A) NRDE-3 retains the ability to bind siRNAs in *nrde-1(−)* animals. FLAG::NRDE-3 co-precipitating RNAs were radiolabeled with ^32^P and analyzed by polyacrylamide gel electrophoresis. (B) NRDE-3 localizes to the nucleus in *nrde-1(−)* animals. Fluorescence microscopy of NRDE-3::GFP in seam cells from ∼L3 animals. (C) The recruitment of NRDE-3 to pre-mRNAs by RNAi is unaffected in *nrde-1(−)* animals. FLAG::NRDE-3 co-precipitating RNAs were converted to cDNA and quantified by qRT-PCR using primers that span exon-intron junctions. Throughout the remainder of this manuscript pre-mRNA levels are measured using exon-intron primer pairs. Data are expressed as a ratio of NRDE-3 precipitating pre-mRNA with or without *lin-15b* RNAi. For wild-type (n = 7,+/− s.e.m.), *nrde-1(−)* (n = 5, +/− s.e.m.), *rrf-1(−)* (n = 5, +/− s.e.m.), *nrde-4(−)* (n = 5, +/− s.e.m.), *rde-1(−)* (n = 3, +/− s.e.m.). Below, western blot detecting FLAG::NRDE-3 verified similar levels of NRDE-3 were Immuno-Precipitated (IP'ed) from each strain and for each condition. (D) NRDE-1 associates with pre-mRNAs that have been targeted by RNAi. NRDE-1 co-precipitating RNAs were converted to cDNA and quantified by qRT-PCR. Data are expressed as a ratio of NRDE-1 co-precipitating pre-mRNAs with or without indicated RNAi. Samples exposed to *dpy-28* RNAi or *lin-15b* RNAi were probed with primers targeting either *dpy-28* pre-mRNA or *lin-15b* pre-mRNA. For *dpy-28* RNAi (n = 3, +/− s.e.m.), *lin-15b* RNAi (n = 5, +/− s.e.m.). For *lin-15b* RNAi, one data point is also shown in panel E and is marked with * in both panels. (E) NRDE-1 association with pre-mRNAs requires NRDE-2 and NRDE-3. FLAG::NRDE-1 co-precipitating pre-mRNAs were converted to cDNA and quantified by qRT-PCR. Data are expressed as a ratio of co-precipitating *lin-15b* pre-mRNA with or without *lin-15b* RNAi. Control (n = 5, +/− s.e.m., data point represented by triangle n = 1), *nrde-2(−)* (n = 3, +/− s.e.m.), *nrde-3(−)* (n = 6, +/− s.e.m.), *nrde-4(−)* (n = 5, +/− s.e.m.), n = 1 for pooled extracts. This experiment was performed in an *nrde-1(gg088)* background. Below, western blot of FLAG::NRDE-1 verified similar amounts of NRDE-1 were IP'ed.

The Ago protein NRDE-3 binds siRNAs in the cytoplasm and transports these siRNAs to the nucleus to facilitate nuclear RNAi [15]. NRDE-3 can bind small RNAs generated from exogenously provided dsRNAs, which are termed exogenous (exo) siRNAs. NRDE-3 also associates with endogenously expressed small RNAs termed endo-siRNAs [15]. NRDE-3 shuttles siRNAs from the cytoplasm to the nucleus; NRDE-3 localizes to the nucleus when bound to either endo or exo-siRNAs, and localizes to the cytoplasm in the absence of these siRNAs [15]. We asked if NRDE-1 was required for NRDE-3/siRNA shuttling. NRDE-3 retained the ability to bind endo-siRNAs in *nrde-1(−)* animals, indicating that *nrde-1* is not required for loading NRDE-3 with siRNAs (Figure 2a). In addition, NRDE-3 remained localized in the nucleus in *nrde-1(−)* animals, indicating that NRDE-1 activity was not required for NRDE-3 shuttling (Figure 2b). These data suggest that NRDE-1 functions downstream of NRDE-3 siRNAs transport. Following exposure to dsRNA, NRDE-3 associates with un-spliced RNAs (pre-mRNA) that exhibit sequence homology to the trigger dsRNA. The association of NRDE-3 with pre-mRNA is dependent upon the ability of NRDE-3 to localize to the nucleus, the ability of NRDE-3 to bind siRNAs, and is restricted to those pre-mRNAs that have been targeted by RNAi [15]. Thus, siRNAs direct NRDE-3 to associate with pre-mRNAs. To test the idea that NRDE-1 functions downstream of NRDE-3 shuttling, we asked if NRDE-1 was required for the association of NRDE-3 with pre-mRNA in response to RNAi. We performed NRDE-3 RNA Immuno-Precipitation (RIP) and found that, in response to *lin-15b* RNAi, NRDE-3 retained the ability to bind *lin-15b* pre-mRNA in *nrde-1(−)* animals, indicating that NRDE-1 functions downstream of NRDE-3/pre-mRNA association during nuclear silencing events (Figure 2c).

Our genetic screen identified nine alleles of the gene *rrf-1* (Figure 1a, Table S1). *rrf-1* encodes one of four *C. elegans* RNA-dependent RNA Polymerases (RdRPs) [34]. We sought to position *rrf-1* in the nuclear RNAi pathway. In animals lacking RRF-1, NRDE-3 binds fewer small RNAs, suggesting that RRF-1 may generate the small RNAs bound by NRDE-3 [15]. Consistent with this idea, in *rrf-1(−)* animals, NRDE-3/*lin-15b* pre-mRNA association was reduced relative to *rrf-1(+)* animals, indicating that RRF-1 acts upstream of NRDE-3/pre-mRNA association during nuclear silencing (Figure 2c). Taken together, these data indicate that our genetic screen is identifying components of the Nrde pathway that function both upstream and downstream of NRDE-3-mediated siRNA transport.

### NRDE-1 is recruited to pre-mRNAs in response to RNAi

We asked if NRDE-1 was recruited to pre-mRNA following RNAi. We performed NRDE-1 RNA Immuno-Precipitation (RIP) experiments in animals exposed to *lin-15b* dsRNA. *lin-15b* RNAi induced a ∼30–70× enrichment in un-spliced *lin-15b* RNA that co-precipitated with FLAG::NRDE-1 (Figure 2d, 2e). The *dpy-28* gene encodes a subunit of the *C. elegans* dosage compensation complex [35]. We tested if *dpy-28* dsRNA would induce NRDE-1-*dpy-28* pre-mRNA association. Following *dpy-28* RNAi, NRDE-1 associated with *dpy-28* pre-mRNA (Figure 2d). Finally, *dpy-28* or *lin-15b* RNAi did not result in enrichment of NRDE-1 with *lin-15b* or *dpy-28* pre-mRNA, respectively, indicating that the association of NRDE-1 with pre-mRNA (induced by RNAi) is sequence specific (Figure 2d). We were concerned that NRDE-1 might associate with pre-mRNA targets, *in vitro*, during sample preparation. To address this issue we pooled extracts from animals exposed to *lin-15b* dsRNA, and extracts from NRDE-1::GFP expressing animals not exposed to *lin-15b*, dsRNA and failed to detect an association of NRDE-1 with *lin-15b* pre-mRNA, indicating that NRDE-1/pre-mRNA interactions likely occurs *in vivo* (Figure 2e). Taken together, these data show that NRDE-1 associates with pre-mRNAs that have been targeted by RNAi.

NRDE-1 co-precipitating pre-mRNA was enriched for RNA sequences encoded at, or near, the site of RNAi- relative to sequences encoded 5′ or 3′ to the site of RNAi (Figure 2e). We have previously shown that NRDE factors fail to associate with pre-mRNA sequences encoded 3′ to the site of RNAi due to RNAi-mediated inhibition of transcription elongation [16]. We investigated the apparent lack of pre-mRNA sequences encoded 5′ to the site of RNAi and found that, while the NRDE factors fail to associate with un-spliced RNA 5′ to the site of RNAi, the Nrdes do associate with spliced RNA 5′ to the site of RNAi (Figure S3). Splicing is thought to occur co-transcriptionally [29]. Therefore, the apparent lack of NRDE-1/pre-mRNA association 5′ to sites of RNAi may be due to co-transcriptional splicing of nascent transcripts.

We investigated the genetic requirements of NRDE-1/pre-mRNA association. In *nrde-2(−)* animals, RNAi failed to induce an association of NRDE-1 with pre-mRNA (Figure 2e). In addition, ∼10× less *lin-15b* pre-mRNA co-precipitated with NRDE-1 in *nrde-3(−)* animals than in *nrde-3(+)* animals (Figure 2e). We conclude that the recruitment of NRDE-1 to pre-mRNAs by small RNAs requires NRDE-2 and is largely dependent upon NRDE-3 (see discussion).

### NRDE-1 promotes RNAi-directed Histone 3 Lysine 9 methylation

**Figure 3:**
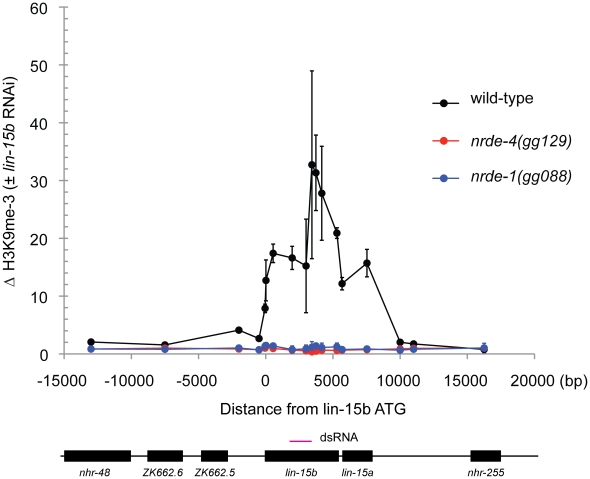
NRDE-1 is required for RNAi-directed H3K9 methylation. Chromatin Immunoprecipitation (ChIP) with anti-H3K9me3 (Upstate, 07-523) was performed on extracts derived from embryos of animals exposed to +/− *lin-15b* RNAi. Co-precipitating H3K9me3 DNA was quantified with qRT-PCR and data are expressed as ratios of samples exposed to *lin-15b* RNAi or no RNAi (n = 3 +/− s.d).

In plants and *S. pombe* small RNAs direct the methylation of Histone 3 Lysine 9 (H3K9me). Histone methylation results from small RNA-mediated recruitment of histone methyltransferase enzymes to genomic sites exhibiting sequence homology to small RNAs [5]. RNAi also directs H3K9 methylation in *C. elegans* [16]. *nrde-2* is required for RNAi-mediated H3K9 methylation in *C. elegans* [16]. The mechanism by which the *C. elegans* Nrde pathway mediates H3K9 methylation is unknown. We conducted H3K9me Chromatin Immuno Precipitation (ChIP) to determine if NRDE-1 was required to link small RNAs to H3K9 methylation. *lin-15b* RNAi induced a ∼30× increase in H3K9me marks at the *lin-15* locus (Figure 3). In *nrde-1(−)* animals, however, *lin-15b* RNAi had no effect on the methylation status of chromatin at the *lin-15b* gene (Figure 3). We conclude that NRDE-1 is required to link small RNAs to H3K9 methylation at a genomic site that has been targeted by RNAi.

### RNAi directs NRDE-1 to associate with chromatin

**Figure 4:**
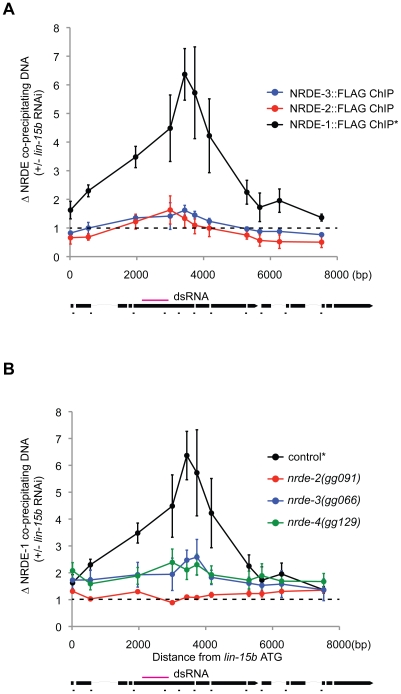
NRDE-1 associates with chromatin at genomic loci targeted by RNAi. (A) FLAG::NRDE co-precipitating DNA was quantified with qRT-PCR. Data are expressed as ratios of NRDE associated DNA with or without *lin-15b* RNAi. Data was normalized to input. NRDE-1 (n = 4, +/− s.e.m.), NRDE-2 (n = 3, +/− s.e.m.), NRDE-3 (n = 3 +/− s.e.m.) * indicates the same FLAG::NRDE-1 ChIP data are shown in panel A and B. (B) Genetic requirements for RNAi-driven NRDE-1/chromatin association. FLAG::NRDE-1 co-precipitating DNA from indicated genetic backgrounds was quantified with qRT-PCR. Data are expressed as ratios of NRDE-1 co-IPed DNA with or without *lin-15b* RNAi. Control (n = 4, +/− s.e.m.), for all other strains (n = 3,+/− s.e.m.).

We asked if the NRDE factors themselves might become associated with chromatin in response to RNAi. In order to address this question, we performed NRDE-1/2/3 ChIP experiments before or after exposure of animals to dsRNA. In response to *lin-15b* RNAi, we did not detect any significant increase in the association of NRDE-2 or NRDE-3 with chromatin at the *lin-15b* gene (Figure 4a). Interestingly, NRDE-1 precipitated ∼6× more *lin-15b* DNA following *lin-15b* RNAi (Figure 4a). In *nrde-2(−)* and *nrde-3(−)* animals, *lin-15b* RNAi failed to trigger an increase in *lin-15b* DNA that co-precipitated with NRDE-1 (Figure 4b). We conclude that NRDE-1 is able to IP chromatin of a gene that has been targeted by RNAi, and that the association of NRDE-1 with chromatin requires NRDE-2 and NRDE-3. It is possible that the ability of NRDE-1 to co-precipitate with chromatin may occur as an indirect consequence of NRDE-1/pre-mRNA interactions. To address this issue, we turned our attention to *nrde-4*.

### NRDE-4 is required for RNAi-directed recruitment of NRDE-1 to chromatin

We mapped and cloned *nrde-4* (Figure S4). *nrde-4* is predicted to encode a protein containing 788 amino acids [33]. Database searches revealed that *nrde-4* is conserved within other nematode species, but not in other species. *nrde-4* encodes a predicted bipartite nuclear localization signal (NLS) and no other obvious protein domains (Figure S4). NRDE-4 is required for silencing nuclear localized RNAs (Table 1), for linking small RNAs to the inhibition of transcription (Figure 1b), and for linking small RNAs to H3K9 methylation (Figure 3). Interestingly, the recruitment of NRDE-1 (and NRDE-2/3) to pre-mRNA was largely unaffected in animals lacking NRDE-4 (Figure 2c, 2e, Figure S5). NRDE-4 was, however, required for recruitment of NRDE-1 to chromatin in response to RNAi (Figure 4b). These data indicate that NRDE-4 functions downstream of NRDE-1/2/3/pre-mRNA interactions during nuclear RNAi. These data also demonstrate that the ability of NRDE-1 to associate with chromatin is dissociable from the ability of NRDE-1 to associate with pre-mRNA, supporting the idea that NRDE-1 associates with chromatin at genomic sites targeted by RNAi.

### Endo-siRNAs direct NRDE-dependent H3K9 methylation

**Figure 5:**
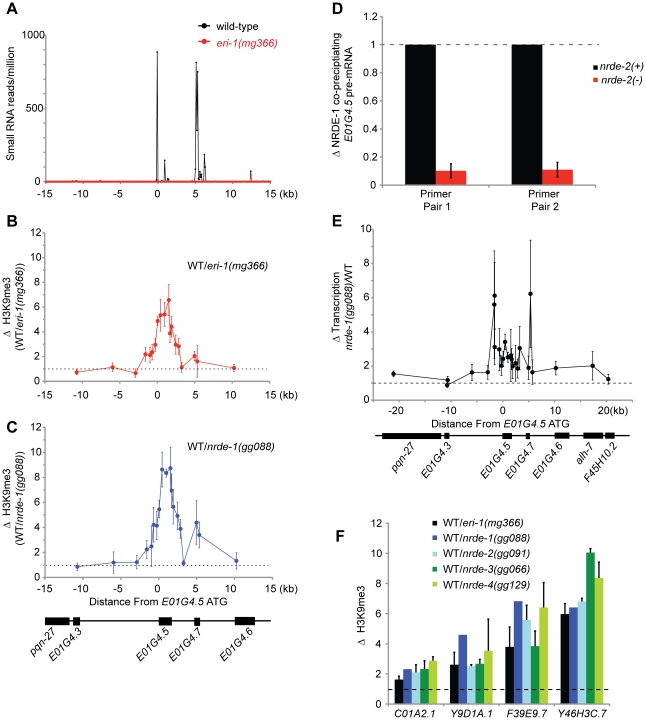
Endo-siRNAs promote H3K9 methylation. (A) *e01g4.5* is an endo-siRNA target. Small RNAs cloned from wild type or *eri-1(mg366)* L4 larval animals [36] were counted in 100 bp non-overlapping windows across a 30 kb region surrounding *e01g4.5*. Small RNAs were normalized to total sequenced small RNAs from each sample. (B–C) *eri-1(−)* and *nrde-1(−)* animals are depleted for H3K9me3 at *e01g4.5*. H3K9me3 ChIPs were performed in wild-type (WT), *eri-1(mg366)*, and *nrde-1(gg088)* animals. Data are represented as ratios of H3K9me3 co-precipitating DNA in WT/*eri-1(mg366)* (B) or WT/*nrde-1(gg088)* (C). Data in B and C are normalized to ChIPed *eft-3* DNA (n = 3, +/− s.d.). (D) NRDE-1 associates with the pre-mRNA of an endo-siRNA target. Co-precipitating FLAG::NRDE-1 RNAs were isolated from +/− *nrde-2* animals, converted to cDNA, and quantified with qRT-PCR. Data are expressed as ratios of NRDE-1 associated *e01g4.5* pre-mRNA +/− *nrde-2* (n = 2, +/− s.d.). (E) Endo-siRNAs inhibit transcription. NRO transcription analysis from wild-type and *nrde-1* mutant animals. Data are represented as a ratio of transcription in *nrde-1(−)*/WT (n = 4, +/− s.e.m.). The genomic region surrounding *e01g4.5* is depicted below the graph. (F) endo-siRNAs direct H3K9me marks at genomic loci homologous to endo-siRNAs. H3K9me3 ChIPs were performed in wild-type (WT) or animals of the indicated genotypes. Data are expressed as ratios of H3K9me co-precipitating DNA in WT/indicated genotype. *eri-1(−)* (n = 3 +/− s.d.), *nrde-1(−)* (n = 1) , *nrde-2(−)* (n = 2, +/− s.d.), *nrde-3(−)* (n = 2, +/− s.d.), *nrde-4(−)* (n = 3, +/− s.d.).

*C. elegans* express at least three types of endogenous small RNAs; the microRNAs, the piRNAs, and the endo-siRNAs. A sub-set of the endo-siRNAs requires ERI-1 for their expression [36], [37]. NRDE-3 associates with the ERI-1-dependent endo-siRNAs, but not the other classes of endogenous small RNAs [15], [37]. Five lines of evidence cumulatively argue that ERI-1 dependent endo-siRNAs are able to direct the deposition of H3K9me marks in *C. elegans*. First, in animals that fail to express endo-siRNAs H3K9me marks are depleted at genomic regions exhibiting sequence complementarity to endo-siRNAs. For instance, *e01g4.5* siRNAs are amongst the most abundant endo-siRNAs expressed in *C. elegans* [36]. *eri-1(−)* animals do not express *e01g4.5* endo-siRNAs ([36], [37] and Figure 5a). We conducted H3K9me ChIP and detected a ∼6× depletion of H3K9me marks at the *e01g4.5* gene in *eri-1(−)* animals (Figure 5b). The changes in H3K9me marks were restricted to genomic regions exhibiting homology to endo-siRNAs; surrounding genomic regions, which are not homologous to known small regulatory RNAs, did not exhibited altered H3K9me marks (Figure 5b). Second, in *nrde-1/2/3/4* mutant animals we observed a similar localized depletion of H3K9me marks at *e01g4.5* (Figure 5c and Figure S6). Third, the *e01g4.5* pre-mRNA was over-expressed 2–5× in *eri-1* and *nrde-1/2/3/4* mutant animals ([15], Figure S7, and data not shown). Fourth, we performed NRDE-1 RIP and quantified the amount of *e01g4.5* pre-mRNA that co-precipitated with NRDE-1. We conducted this experiment in both *nrde-2(+)* and *nrde-2(−)* animals as NRDE-2 is required for NRDE-1 recruitment to pre-mRNAs in response to feeding RNAi. We found that NRDE-1 associated with ∼5× more *e01g4.5* pre-mRNA in *nrde-2(+)* animals than in *nrde-2(−)* animals (Figure 5d). These data suggest that NRDE-1 can associate with pre-mRNAs that are homologous to endo-siRNAs, and that this process depends upon components of the Nrde pathway. Five, we detected a subtle and complex, yet reproducible, increase in transcription at the *e01g4.5* gene in *eri-1* and *nrde-1/2/4* mutant animals (Figure 5e and Figure S8). Taken together, these data indicate that *e01g4.5* endo-siRNAs are able to direct chromatin modification in *C. elegans* and that this process requires the Nrde pathway.

Lastly, we investigated the generality of small RNA-mediated chromatin regulation in *C. elegans*. We queried seven additional genomic sites that exhibit sequence homology to *eri-1*-dependent endo-siRNAs. At four of these loci H3K9me marks were depleted in *eri-1* and *nrde-1/2/3/4* animals (Figure 5f). At three of these loci, no significant differences in H3K9me marks were observed. We conclude that Nrde-dependent endogenous small RNA-mediated chromatin modification occurs at multiple loci in *C. elegans*.

## Discussion

Here we report that small RNAs are necessary and sufficient to direct chromatin modification in *C. elegans*. We show that a class of endogenous small RNAs, termed the endo-siRNAs, direct H3K9 methylation at discrete genomic loci, and that this process requires the Nrde pathway. Finally, we identify two novel nuclear RNAi factors including NRDE-1, which we show is recruited to pre-mRNAs and chromatin by RNAi, and is required to link small RNAs to chromatin regulation.

### Hierarchical assembly of nrde factors on nascent RNAs

**Figure 6:**
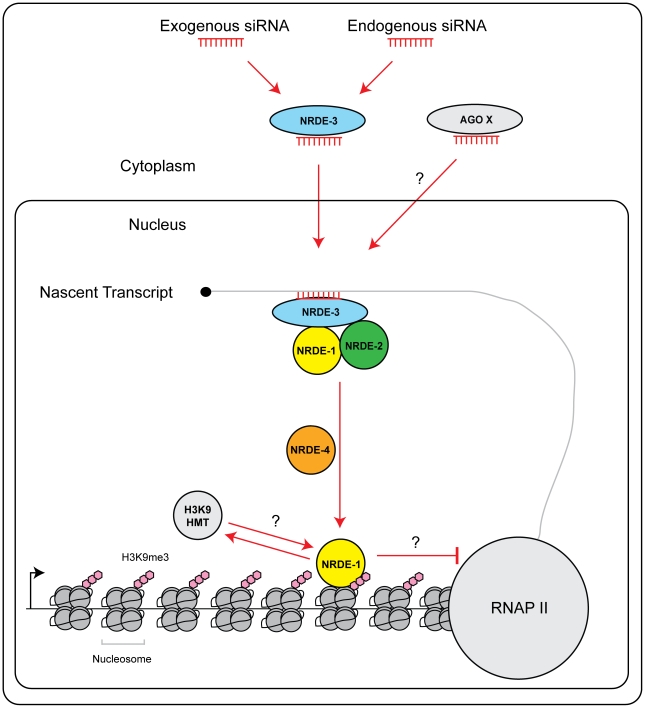
Model of NRDE pathway. siRNAs (from either an exogenous or endogenous source) are bound by the Ago NRDE-3 in the cytoplasm and escorted into the nucleus. Once in the nucleus, NRDE-3/siRNA complexes bind nascent transcripts synthesized by RNAP II. NRDE-3/siRNA complexes recruit NRDE-2, and NRDE-2/3 recruit NRDE-1 to these nascent transcripts. NRDE-1 is deposited on chromatin in a NRDE-4 dependent manner. An unknown histone methyltransferase (HMT) catalyzes the methylation of histone 3 lysine 9 (H3K9me3). H3K9me marks may facilitate recruitment of NRDE-1 to chromatin. Alternatively, NRDE-1 may recruit an H3K9 methyltransferase to sites of RNAi. Together, H3K9me marks and NRDE-1 inhibit RNAP II elongation. Additional small RNAs and Argonaute proteins (Ago X) may engage NRDE-1/2/4 to direct chromatin modifications.

In *S. pombe* silencing factors assemble upon nascent transcripts during nuclear RNAi [3]. Here, we present evidence that nascent transcripts serve a similar role in *C. elegans*. The Ago NRDE-3 is guided to nascent transcripts via base pairing between NRDE-3 bound siRNAs and nascent transcripts [15]. In *nrde-1* mutant animals, NRDE-3 can still associate with the target pre-mRNA, but nuclear silencing does not occur (Figure 2c). Thus, NRDE-3 bound siRNAs provide the information of *where* to silence, but additional downstream factors, such as NRDE-1, are required for silencing to occur. NRDE-3 is required for recruitment of NRDE-2 to pre-mRNA in response to RNAi [16]. NRDE-3 and NRDE-2 are required for the recruitment of NRDE-1 to pre-mRNAs in response to RNAi (Figure 2e). Thus, we propose that the NRDE factors assemble in a hierarchical manner on pre-mRNA; NRDE-3 identifies pre-mRNAs, and in association with NRDE-2, recruits NRDE-1 to pre-mRNAs that have been targeted by RNAi (Figure 6).

### NRDE-1 and NRDE-4 in nuclear RNAi

What is the role of NRDE-4 in nuclear RNAi? Our preliminary investigation has shown that: NRDE-4 is required to link small RNAs to transcription and chromatin regulation. Interestingly, we find that NRDE-4 is not required for small RNA-directed NRDE-1/pre-mRNA association, but is, required for the recruitment of NRDE-1 to chromatin. Therefore, it seems reasonable to speculate that one role of NRDE-4 during nuclear RNAi may be to load/stabilize NRDE-1 on chromatin, following the recruitment of NRDE-1 to pre-mRNAs by NRDE-2/3 (Figure 6).

What is the role of NRDE-1 in nuclear RNAi? In response to RNAi, NRDE-1 co-precipitates with both pre-mRNAs and chromatin. We did not detect an association of NRDE-3 or NRDE-2 with chromatin despite the fact that NRDE-2/3 are able, like NRDE-1, to associate with pre-mRNA in response to RNAi (Figure 4a). These data hint that NRDE-1 may possess a chromatin associating property not exhibited by NRDE-2/3. We considered the possibility that NRDE-1 might IP chromatin indirectly via pre-mRNA/RNAP II intermediates. However, we found that in *nrde-4(−)* animals, NRDE-1 is recruited to pre-mRNAs by RNAi, but does not become associated with chromatin (Figure 2e and Figure 4b). These data demonstrate that the RNA and chromatin associating properties of NRDE-1 can be separated. Additionally, we find that NRDE-1 association with RNA occurs predominantly 5′ to the site of RNAi, whereas NRDE-1 association with chromatin occurs predominantly 3′ to the site of RNAi (Figure 2e and Figure 4a). Taken together, these data argue that NRDE-1 associates with chromatin in response to RNAi, and that NRDE-1 interacts with pre-mRNAs first and chromatin second during nuclear silencing processes (Figure 6). The question then becomes; what is the role of NRDE-1 at chromatin?

### H3K9me and nuclear RNAi

Here we show that small RNAs promote H3K9 methylation in *C. elegans*. We show that experimentally introduced small RNAs are sufficient to direct H3K9me marks at genomic sites targeted by RNAi. We also show that small RNAs are necessary to establish H3K9me marks; in animals that fail to express endogenous siRNAs, H3K9me marks are depleted at genomic sites homologous to endo-siRNAs. In *S. Pombe*, the RNAi machinery directs H3K9 methylation at pericentromeric repeats via recruitment of the H3K9 methyltransferase Clr4 to pre-mRNAs exhibiting homology to pericentromeric siRNAs [5]. Interestingly, fungi lacking H3K9me, due to loss of Clr4, fail to express abundant pericentromeric siRNAs [38]. Thus, H3K9me and the RNAi machinery are thought to comprise a self-reinforcing loop that facilitates heterochromatin formation at pericentromeric regions in *S. pombe* [39], [40]. We find that, in *C. elegans*, RNAi directs both H3K9 methylation and the association of NRDE-1 with chromatin. These data hint that *C. elegans* may employ a similar strategy as *S. pombe* for establishing heterochromatin; *e.g*. RNAi promotes H3K9 methylation and H3K9 methylation may help recruit components of the RNAi machinery, such as NRDE-1, to chromatin. In order to test this model, the *C. elegans* methyltransferase(s) responsible for depositing H3K9me marks in response to RNAi will need to be identified.

### H3K9me and transcription

We find that H3K9me marks become distributed throughout a gene that has been targeted by RNAi (Figure 3). These data raise several interesting questions. First, how do H3K9me marks spread from the site of RNAi, and how are these marks prevented from spreading into adjacent genes? A simple model posits that the deposition of H3K9me marks (directed by small RNAs) is coupled to transcription in *C. elegans*. In other words, the act of transcription may alter chromatin in such a way as to permit (and limit) H3K9me spreading. Another question that arises is; what is the connection between H3K9 methylation and RNAP II transcription in *C. elegans*? We show that both endo-siRNAs and exo-siRNAs direct H3K9 methylation, which correlates with decreases in transcription. These data are consistent with the established repressive role of H3K9 methylation on transcription [8]. We find that RNAi-directed H3K9me marks peak 3′ to sites of RNAi (Figure 3). In addition, we find that NRDE-1 associates with chromatin predominantly 3′ to sites of RNAi (Figure 4a), and RNAP II transcription is inhibited by RNAi predominantly 3′ to the site of RNAi (Figure 1b). Therefore, H3K9me marks and NRDE-1 may contribute to the inhibition of RNAP II elongation by small RNAs (Figure 6). It should be noted, however, that while H3K9me marks peak 3′ to sites of RNAi, we observe H3K9 methylation throughout genes targeted by RNAi, hinting that H3K9me marks alone may not be sufficient to inhibit RNAP II transcription in *C. elegans* (Figure 3).

### Why nuclear RNAi?

In *S. pombe* small RNAs primarily target repetitive genomic elements. RNAi-directed heterochromatization at pericentromeric repeats permits efficient segregation of chromosomes during meiosis [41]. In plants, small RNAs silence genomic regions enriched in transposons, pericentromeric regions, and rRNA genes [1]. Here we show that ERI-1-dependent endo-siRNAs direct the establishment of heterochromatic marks on chromatin. The biological role(s) of this small RNA-mediated chromatin regulation in *C. elegans* is unknown. The ERI-1-dependent endo-siRNAs are anti-sense to several hundred cellular mRNAs [36]. In general, these mRNAs appear to be poorly conserved and repetitive, hinting that these mRNAs may represent the products of dead and dying genes [42]. The purpose of nuclear RNAi may be to prevent expression of these dysfunctional genes. Alternatively, these mRNAs may simply serve as templates for the creation of small RNAs, which, in turn, regulate chromatin dynamics.

There are 26 Agos encoded in the worm genome, in addition to *nrde-3* [43]. We have detected pleiotropic fertility defects exhibited by *nrde-1/2/4(−)*, but not *nrde-3(−)*, animals, hinting that other Ago proteins and, perhaps, other types of small RNAs, may engage NRDE-1/2/4 to promote H3K9 methylation during development (Figure S9). In support of this idea, we find that the recruitment of NRDE-1 to pre-mRNAs and chromatin, in response to RNAi, is not completely abolished in animals harboring null alleles of *nrde-3* (Figure 2e). These data support the idea that other Ago proteins may engage the Nrde pathway to elicit nuclear silencing and chromatin regulation in *C. elegans* (Figure 2e, Figure 6). The identification of these Ago factors and their small RNA partners will be important for unraveling the cellular connections that exist between endogenous small RNAs and chromatin dynamics in metazoans.

## Materials and Methods

### Strains

N2, (YY160) *nrde-1(gg088)*, (YY186) *nrde-2(gg091)*, (YY158) *nrde-3(gg066)*, (YY453) *nrde-4(gg129)*, (GR1373) *eri-1(mg366)*, (YY191) *eri-1(mg366)*; *nrde-1(gg088)*, (YY468) *eri-1(mg366)*; *nrde-4(gg129)*, (YY268) *nrde-1(gg088)*; *ggIS12[nrde-3p::3xflag::gfp::nrde-1]*, (YY464) *nrde-1(gg088)*; *nrde-2(gg091)*; *ggIS12*, (YY459) *nrde-1(gg088)*; *nrde-3(gg066)*; *ggIS12*, (YY462) *nrde-1(gg088)*; *nrde-4(gg129)*; *ggIS12*, (YY174) *ggIS1[nrde-3p::3xflag::gfp::nrde-3]*, (YY225) *rde-1(ne219)*; *ggIS1*, (YY228) *nrde-1(gg088)*; *ggIS1*, (YY454) *nrde-4(gg129)*; *ggIS1*, (YY230) *rrf-1(pk1417)*; *ggIS1*, (YY346) *nrde-2(gg091)*; *ggIS28[nrde-3p::3xflag::gfp::nrde-2]*.

### Construction of plasmids and transgenic strains

For FLAG::GFP::NRDE-1 (referred to as GFP::NRDE-1 when assaying NRDE-1 expression or FLAG::NRDE-1 when referring to NRDE-1 immunoprecipitation or western blotting) the *nrde-1* coding region and predicted 3′UTR were amplified by PCR from genomic N2 DNA and inserted into the pSG082 plasmid 3′ to the *nrde-3p::*3xFLAG::GFP. Low copy integrated transgenes were generated by biolistic transformation [44].

### RNAi

RNAi experiments were conducted as described previously [45]. The *lir-1* and *unc-15* bacterial clones were taken from the Ahringer library [46]. The *lin-15b* clone was described previously [15].

### RNA IP (RIP)

RIPs were performed as described previously [15]. Hypochlorite-isolated embryos were used for all RIPs. FLAG::NRDE-1 and FLAG::NRDE-3 proteins were immuno-precipitated with anti-FLAG M2 antibody (Sigma, A2220).

### Chromatin IP (ChIP)

ChIP experiments were performed as described previously [16]. Hypochlorite-isolated embryos were used for ChIP experiments. Isolated embryos were snap-frozen in liquid-Nitrogen before performing ChIP. FLAG::NRDE-1, FLAG::NRDE-2, and FLAG::NRDE-3 proteins were immuno-precipitated with anti-FLAG M2 antibody (Sigma, A2220). H3K9me3 antibody was from Upstate (07-523).

### Nuclear run on (NRO) assay

NRO was performed as described previously [16]. Hypochlorite-isolated embryos were used for NROs.

### cDNA preparation

RNAs were converted to cDNA by the iScript cDNA Synthesis Kit (Bio-Rad, 170–8890) following the vendor's protocol.

## Supporting Information

Figure S1 Modified genetic screen. We screened for cellular factors that were required for the silencing of nuclear localized RNAs. Wild-type animals expressing ectopic copies of *nrde-3* (NRDE-3::GFP) were mutagenized and exposed to *lir-1* RNAi. The majority of animals exposed to *lir-1* RNAi died due to the silencing of the *lir-1/lin-26* pre-mRNA. Animals that survived *lir-1* RNAi were isolated and subjected to a secondary screen using *pos-1* RNAi. Mutant animals that survived *pos-1* RNAi were discarded, as we anticipate these animals harbor mutations in the upstream and cytoplasmic RNAi machinery. The remaining alleles were assigned to complementation groups.
(TIF)

Figure S2 *nrde-1* functions in a genetic pathway with *nrde-2* and *nrde-3*. *unc-15* RNAi directs a partially penetrant Uncoordinated (Unc) phenotype in control animals. Single and double *nrde* mutant strains were scored for Unc phenotypes in response to *unc-15* RNAi. The number of animals exhibiting a paralysis phenotype and the strength of the paralysis phenotype was scored blinded on an scale from 0–4. The paralysis phenotype of non-blinded *eri-1(mg366)* animals fed *unc-15* RNAi was defined as ‘4’ (100% animals paralyzed), and *eri-1(mg366);rde-1(ne219)* was defined as ‘0’ (0% of animals paralyzed). 10–100 animals were scored in each trial (n≥5). *nrde-1/2/3* mutants are partially suppressed for *unc-15* RNAi-meditated paralysis. *nrde-1;nrde-2*, and *nrde-1;nrde-3* double mutants do not have a synergistic effect on *unc-15* RNAi, suggesting that *nrde-1/-2/-3* function in the same genetic pathway. The genetic background of this experiment was *eri-1(mg366)*.
(TIF)

Figure S3 NRDE factors associate with spliced RNAs encoded 5′ to the site of RNAi. (A) NRDE-1 associates with partially spliced RNAs 5′ to site of RNAi. NRDE-1 co-precipitating RNAs were converted to cDNA and quantified by qRT-PCR using primers that recognize exon-exon splice junctions (spliced RNA). Data are expressed as a ratio of NRDE-1 precipitating RNA with or without *lin-15b* RNAi. This experiment was performed in a *nrde-1(gg088)* background (n = 1). (B) NRDE-2 associates with spliced RNA encoded 5′ to the site of RNAi. NRDE-2 co-precipitating RNAs were converted to cDNA and quantified by qRT-PCR using primers that span splice junctions (spliced RNA) or exon-intron junctions (pre-mRNA). Data are expressed as a ratio of NRDE-2 precipitating RNA with or without *lin-15b* RNAi (n = 3 +/−, s.d.).
(TIF)

Figure S4 Molecular identity of *nrde-4*. (A) *nrde-4* gene structure. Arrows indicate mutant alleles. *gg129*, *gg131*, and *gg132* were identified in screen described in main text and Figure S1. *gg194* was identified in screen described in panel C. (B) Amino acid sequence of NRDE-4. Amino acids in red indicate mutated amino acids in *gg129*, *gg131*, and *gg132*. Amino acids highlighted in yellow encode a putative Nuclear Localization Signal (NLS). (C) Yet another, and hopefully last, screen for factors required for nuclear RNAi. *eri-1(mg366)* animals exposed to *dpy-13* dsRNA exhibit a super-Dpy phenotype. Mutant alleles that suppressed *dpy-13* RNAi-mediated super-Dpy phenotype were selected and subjected to the indicated secondary screens. *gg194* was mapped to a genomic region containing *f45e4.10*. *f45e4.10* was sequenced and the *gg194* lesion was identified.
(TIF)

Figure S5 *nrde-4* acts downstream of NRDE-2/pre-mRNA association. NRDE-2 associates with pre-mRNAs targeted by RNAi in an *nrde-4* independent manner. FLAG::NRDE-2 co-precipitating pre-mRNAs were converted to cDNA and quantified by qRT-PCR. Data are expressed as a ratio of co-precipitating *lin-15b* pre-mRNA with or without *lin-15b* RNAi. Wild-type (n = 2–6, +/− s.d.), *nrde-4(−)* (n = 2, +/− s.d.).
(TIF)

Figure S6 *nrde-2*, *nrde-3*, and *nrde-4* are required for endo-siRNA driven H3K9me3. (A–C) H3K9me3 ChIPs were performed in wild-type (WT), *nrde-2(gg091)*, *nrde-3(gg066)*, or *nrde-4(gg129)* animals. Data are represented as ratios of *e01g4.5* co-precipitating DNA in WT/*nrde*. *nrde-2(−)* (n = 3; +/− s.d.), *nrde-3(−)* (n = 3 +/− s.d.), *nrde-4(−)* (n = 5; +/− s.d.).
(TIF)

Figure S7 *e01g4.5* pre-mRNA is elevated in *nrde-2* and *nrde-4* mutants. RNA from WT and *nrde* mutants was isolated from embryos and converted to cDNA. Primers that span exon-intron junctions were used to quantify *e01g4.5* pre-mRNA using qRT-PCR. Data was normalized to *eft-3* pre-mRNA. WT was defined as 1 (n = 3, +/− s.d.). Experiment was done in a background containing the NRDE-3::FLAG (*ggIS1*) transgene.
(TIF)

Figure S8 Increased transcription of *e01g4.5* in *eri-*1 and *nrde-2/-4* animals. *eri-1* and *nrde-2/-4* are required for transcriptional silencing of *e01g4.5*. Data are represented as a ratio of transcription in mutant/WT. Two different primer pairs in the *e01g4.5* gene were used to quantify transcription. (n = 2–3, +/−s.d.).
(TIF)

Figure S9 A subset of *nrde* mutants have reduced fecundity. *nrde-1/-2/-4* mutants have reduced brood sizes. Brood sizes were counted from individual animals grown at 25°C. (error bars +/− s.d.).
(TIF)

Table S1 A modified genetic screen identifies novel nuclear RNAi factors. We have performed two genetic screens for Nrde factors. The table represents the genes (and number of mutant alleles) identified in each of the two screens. In screen 2, we mutagenized *eri-1(+)* animals expressing additional copies of *nrde-3* in the form of a rescuing and integrated *nrde-3*::*gfp* transgene. As expected, we failed to identify *nrde-3* alleles in screen 2.
(XLSX)
