# Supplementary figures and images for: A Pre-mRNA–Associating Factor Links Endogenous siRNAs to Chromatin Regulation
Source: PLoS Genet. 2011 Aug 25;7(8):e1002249. doi: 10.1371/journal.pgen.1002249 (PMC3161925; doi:10.1371/journal.pgen.1002249)

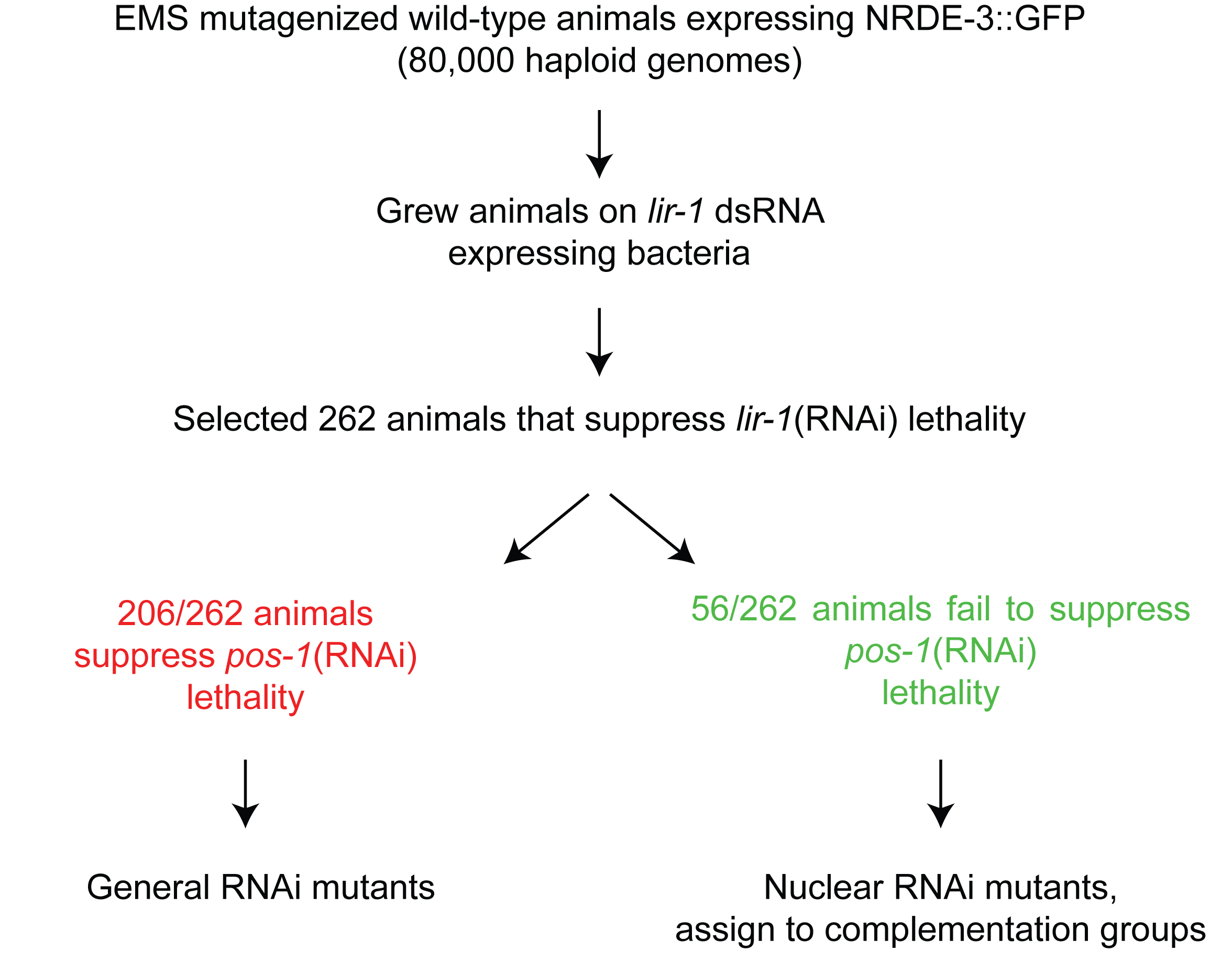

Supplement: Figure S1 — Modified genetic screen. We screened for cellular factors that were required for the silencing of nuclear localized RNAs. Wild-type animals expressing ectopic copies of nrde-3 (NRDE-3::GFP) were mutagenized and exposed to lir-1 RNAi. The majority of animals exposed to lir-1 RNAi died due to the silencing of the lir-1/lin-26 pre-mRNA. Animals that survived lir-1 RNAi were isolated and subjected to a secondary screen using pos-1 RNAi. Mutant animals that survived pos-1 RNAi were discarded, as we anticipate these animals harbor mutations in the upstream and cytoplasmic RNAi machinery. The remaining alleles were assigned to complementation groups. (TIF) [file pgen.1002249.s001.tif]

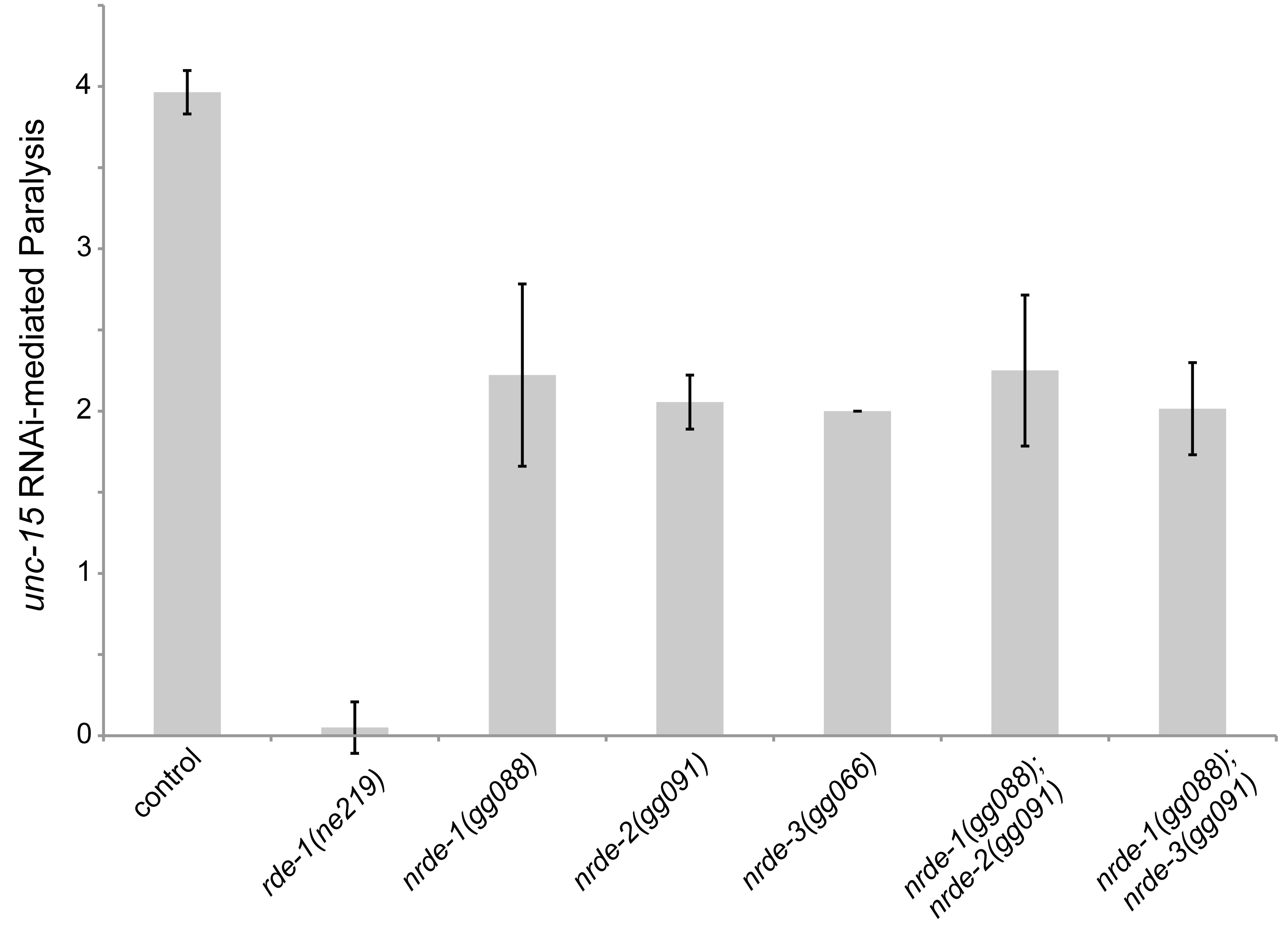

Supplement: Figure S2 — nrde-1 functions in a genetic pathway with nrde-2 and nrde-3. unc-15 RNAi directs a partially penetrant Uncoordinated (Unc) phenotype in control animals. Single and double nrde mutant strains were scored for Unc phenotypes in response to unc-15 RNAi. The number of animals exhibiting a paralysis phenotype and the strength of the paralysis phenotype was scored blinded on an scale from 0–4. The paralysis phenotype of non-blinded eri-1(mg366) animals fed unc-15 RNAi was defined as ‘4’ (100% animals paralyzed), and eri-1(mg366);rde-1(ne219) was defined as ‘0’ (0% of animals paralyzed). 10–100 animals were scored in each trial (n≥5). nrde-1/2/3 mutants are partially suppressed for unc-15 RNAi-meditated paralysis. nrde-1;nrde-2, and nrde-1;nrde-3 double mutants do not have a synergistic effect on unc-15 RNAi, suggesting that nrde-1/-2/-3 function in the same genetic pathway. The genetic background of this experiment was eri-1(mg366). (TIF) [file pgen.1002249.s002.tif]

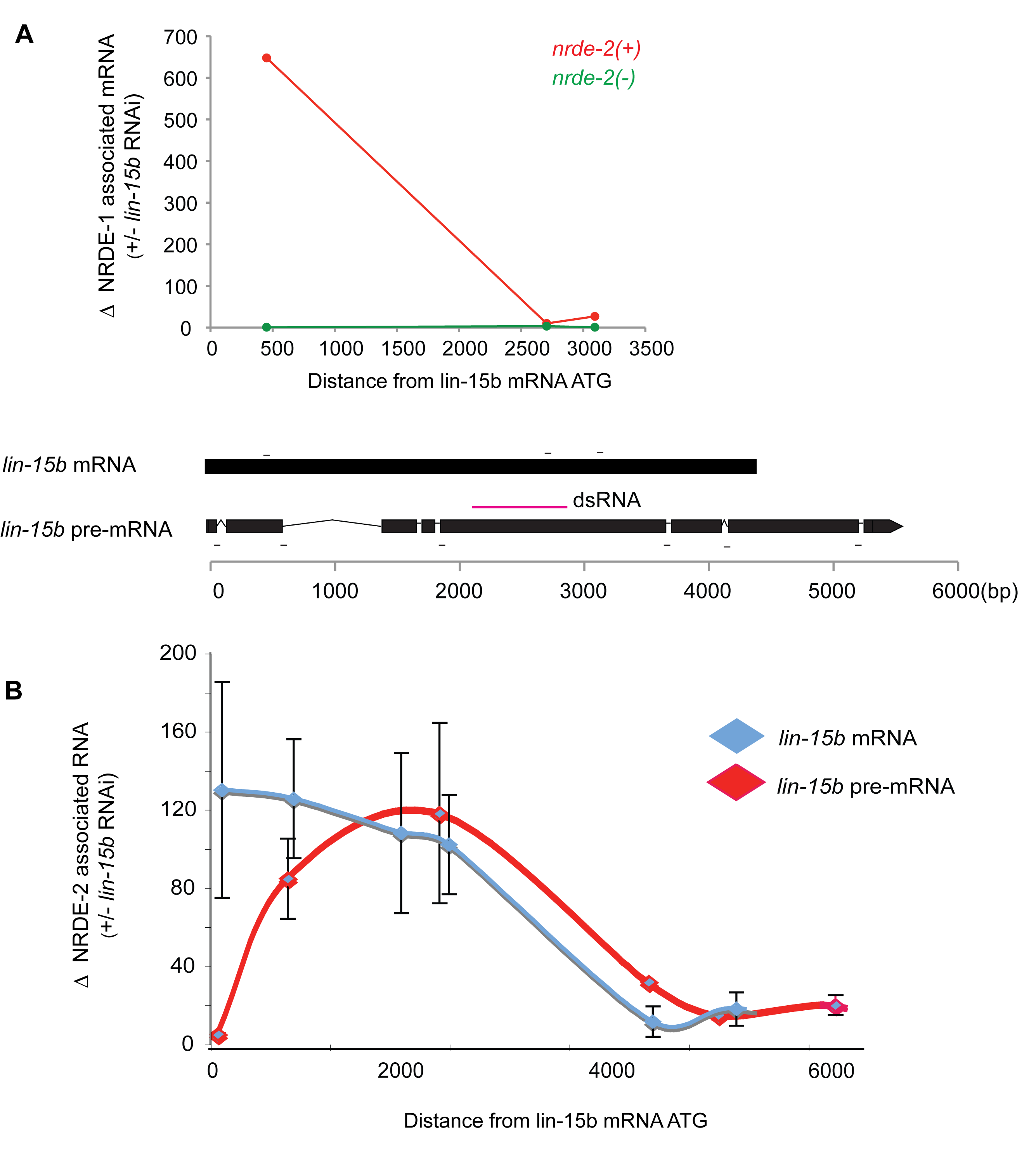

Supplement: Figure S3 — NRDE factors associate with spliced RNAs encoded 5′ to the site of RNAi. (A) NRDE-1 associates with partially spliced RNAs 5′ to site of RNAi. NRDE-1 co-precipitating RNAs were converted to cDNA and quantified by qRT-PCR using primers that recognize exon-exon splice junctions (spliced RNA). Data are expressed as a ratio of NRDE-1 precipitating RNA with or without lin-15b RNAi. This experiment was performed in a nrde-1(gg088) background (n = 1). (B) NRDE-2 associates with spliced RNA encoded 5′ to the site of RNAi. NRDE-2 co-precipitating RNAs were converted to cDNA and quantified by qRT-PCR using primers that span splice junctions (spliced RNA) or exon-intron junctions (pre-mRNA). Data are expressed as a ratio of NRDE-2 precipitating RNA with or without lin-15b RNAi (n = 3 +/−, s.d.). (TIF) [file pgen.1002249.s003.tif]

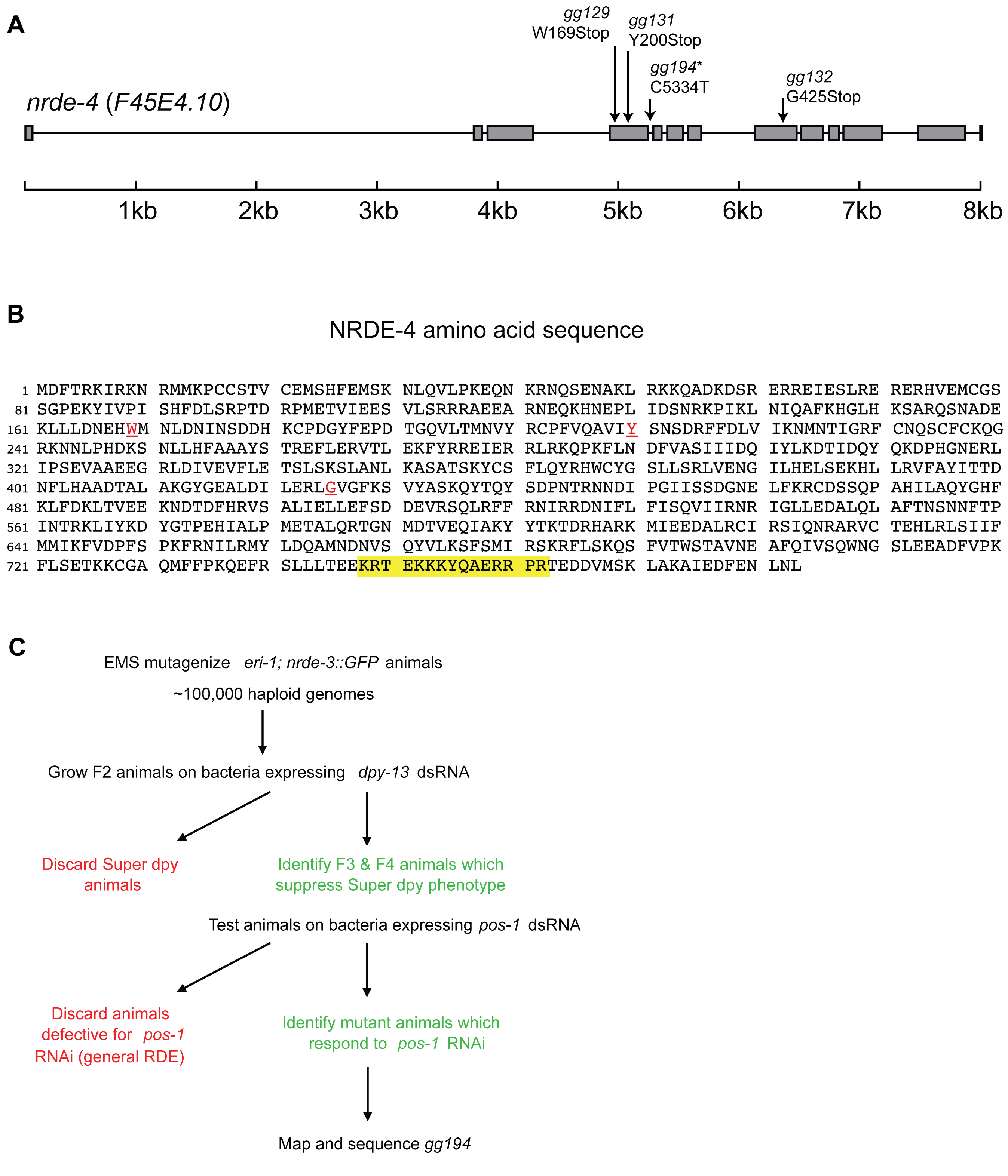

Supplement: Figure S4 — Molecular identity of nrde-4. (A) nrde-4 gene structure. Arrows indicate mutant alleles. gg129, gg131, and gg132 were identified in screen described in main text and Figure S1. gg194 was identified in screen described in panel C. (B) Amino acid sequence of NRDE-4. Amino acids in red indicate mutated amino acids in gg129, gg131, and gg132. Amino acids highlighted in yellow encode a putative Nuclear Localization Signal (NLS). (C) Yet another, and hopefully last, screen for factors required for nuclear RNAi. eri-1(mg366) animals exposed to dpy-13 dsRNA exhibit a super-Dpy phenotype. Mutant alleles that suppressed dpy-13 RNAi-mediated super-Dpy phenotype were selected and subjected to the indicated secondary screens. gg194 was mapped to a genomic region containing f45e4.10. f45e4.10 was sequenced and the gg194 lesion was identified. (TIF) [file pgen.1002249.s004.tif]

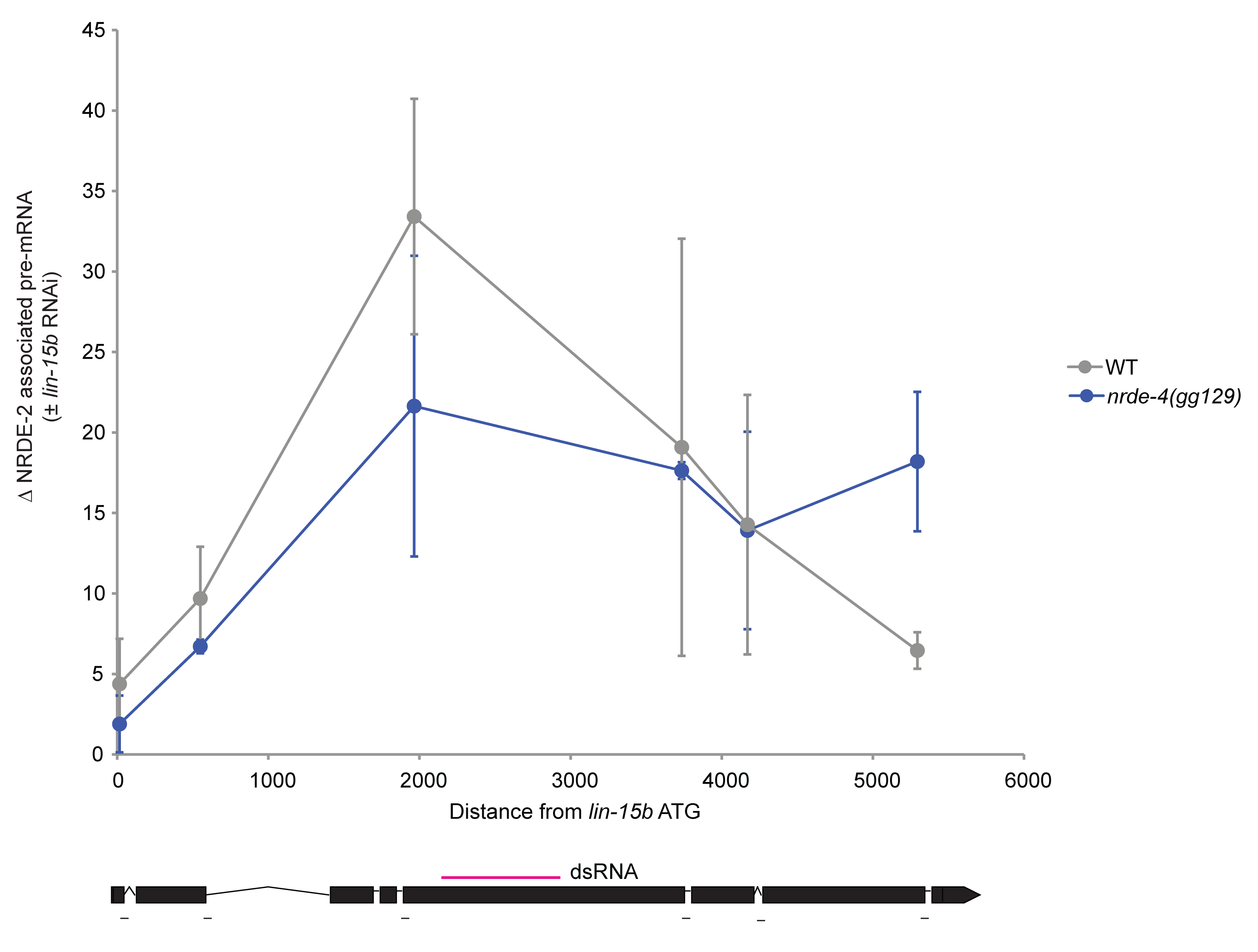

Supplement: Figure S5 — nrde-4 acts downstream of NRDE-2/pre-mRNA association. NRDE-2 associates with pre-mRNAs targeted by RNAi in an nrde-4 independent manner. FLAG::NRDE-2 co-precipitating pre-mRNAs were converted to cDNA and quantified by qRT-PCR. Data are expressed as a ratio of co-precipitating lin-15b pre-mRNA with or without lin-15b RNAi. Wild-type (n = 2–6, +/− s.d.), nrde-4(−) (n = 2, +/− s.d.). (TIF) [file pgen.1002249.s005.tif]

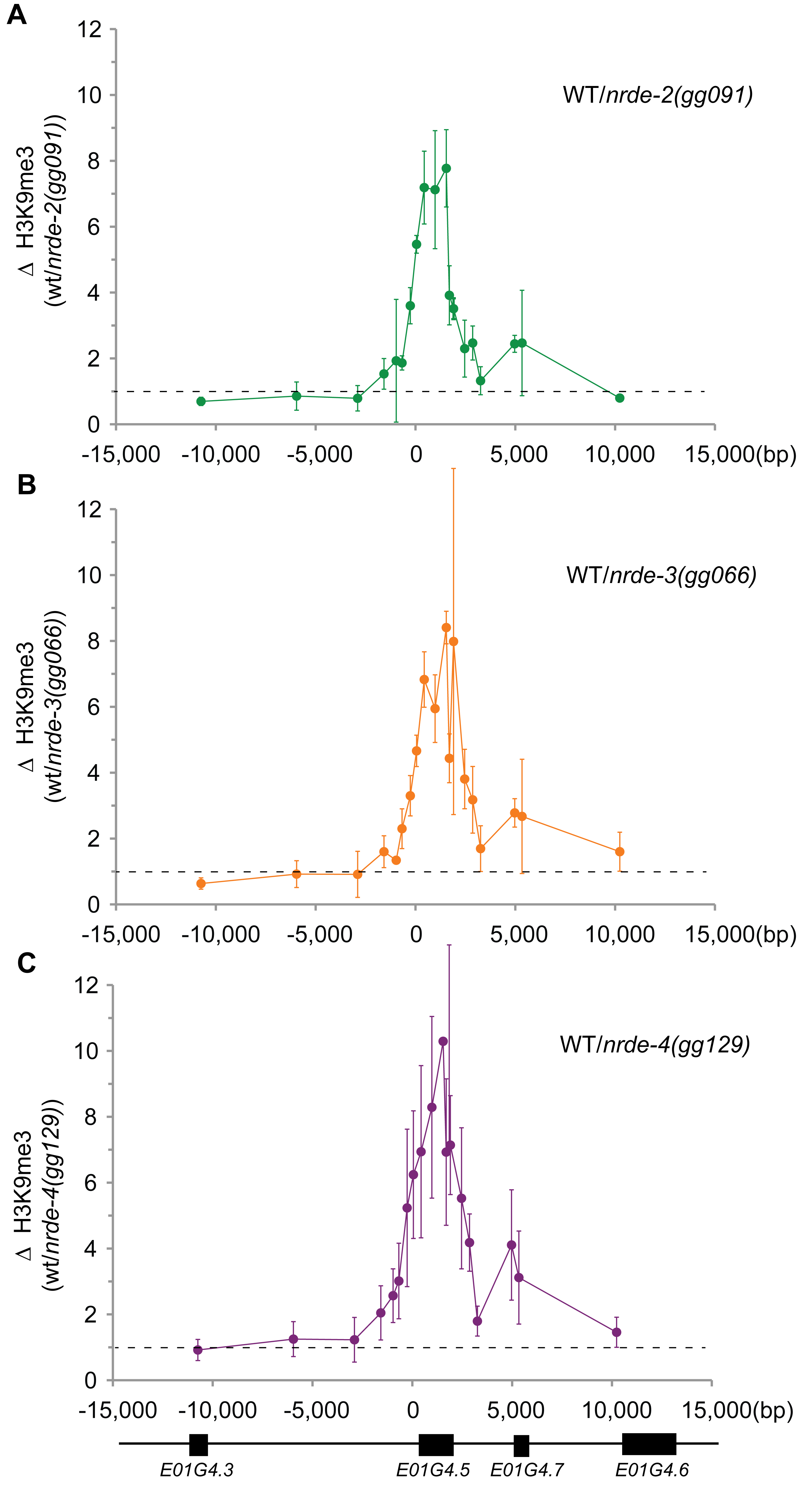

Supplement: Figure S6 — nrde-2, nrde-3, and nrde-4 are required for endo-siRNA driven H3K9me3. (A–C) H3K9me3 ChIPs were performed in wild-type (WT), nrde-2(gg091), nrde-3(gg066), or nrde-4(gg129) animals. Data are represented as ratios of e01g4.5 co-precipitating DNA in WT/nrde. nrde-2(−) (n = 3; +/− s.d.), nrde-3(−) (n = 3 +/− s.d.), nrde-4(−) (n = 5; +/− s.d.). (TIF) [file pgen.1002249.s006.tif]

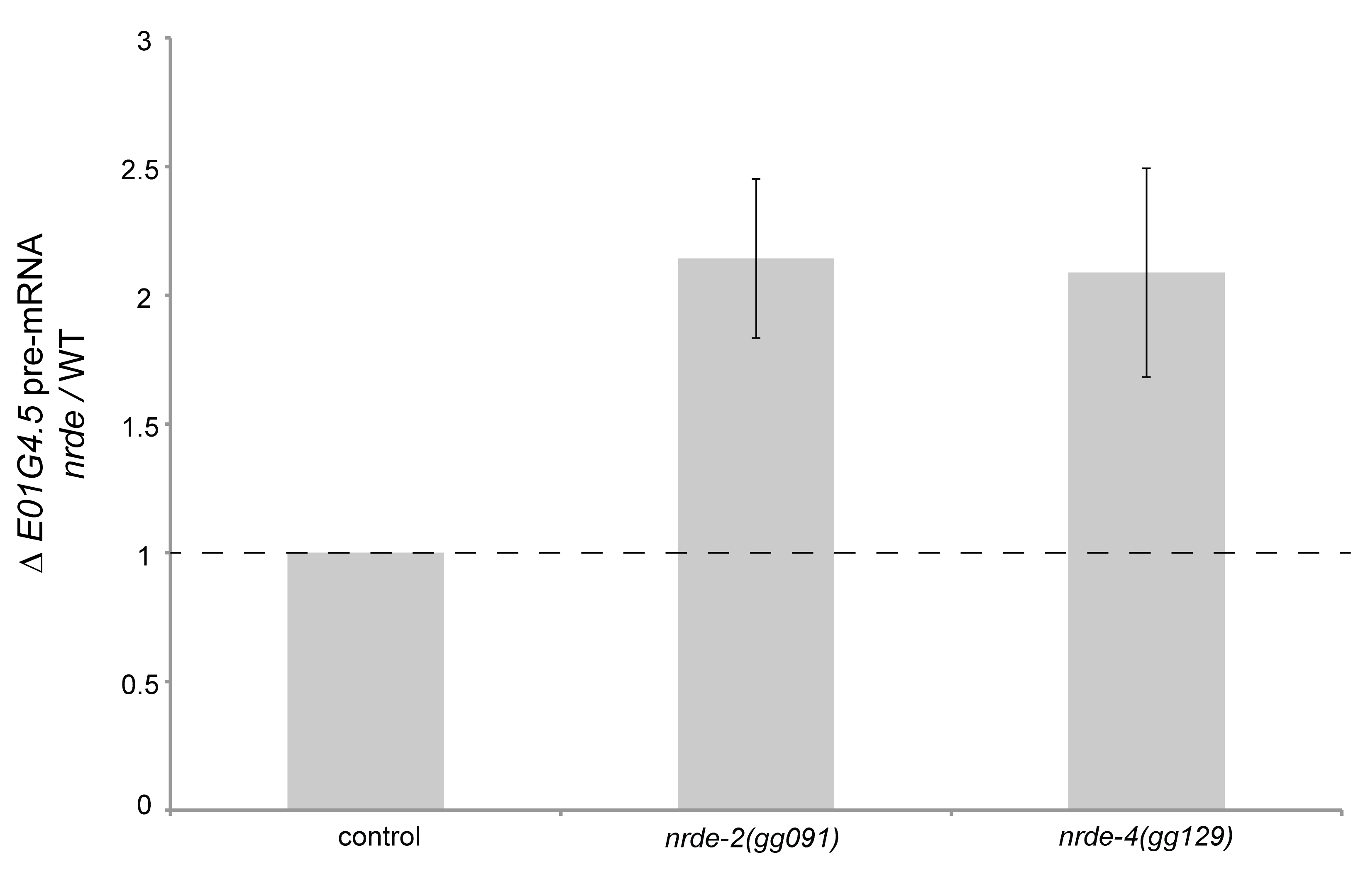

Supplement: Figure S7 — e01g4.5 pre-mRNA is elevated in nrde-2 and nrde-4 mutants. RNA from WT and nrde mutants was isolated from embryos and converted to cDNA. Primers that span exon-intron junctions were used to quantify e01g4.5 pre-mRNA using qRT-PCR. Data was normalized to eft-3 pre-mRNA. WT was defined as 1 (n = 3, +/− s.d.). Experiment was done in a background containing the NRDE-3::FLAG (ggIS1) transgene. (TIF) [file pgen.1002249.s007.tif]

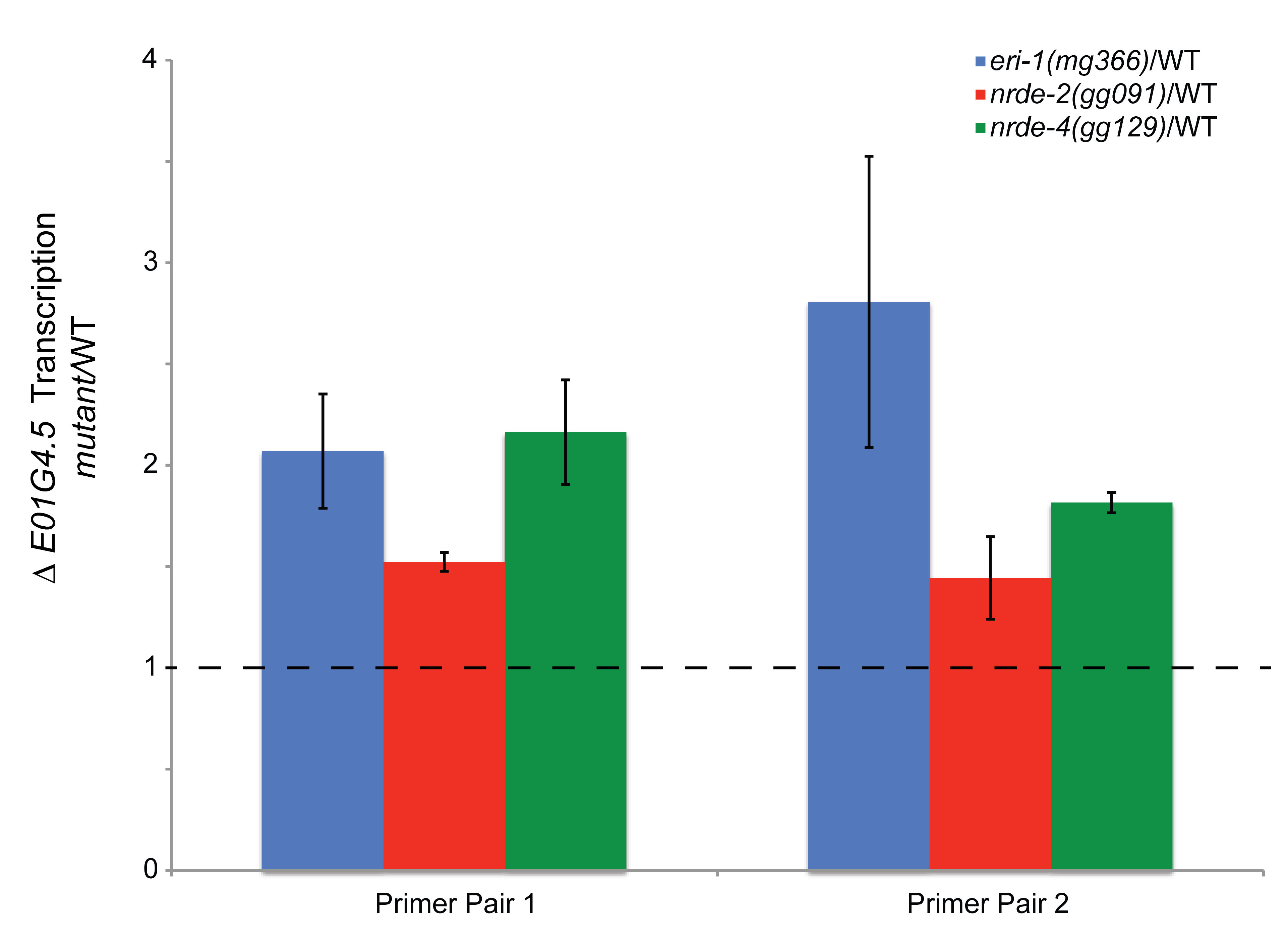

Supplement: Figure S8 — Increased transcription of e01g4.5 in eri-1 and nrde-2/-4 animals. eri-1 and nrde-2/-4 are required for transcriptional silencing of e01g4.5. Data are represented as a ratio of transcription in mutant/WT. Two different primer pairs in the e01g4.5 gene were used to quantify transcription. (n = 2–3, +/−s.d.). (TIF) [file pgen.1002249.s008.tif]

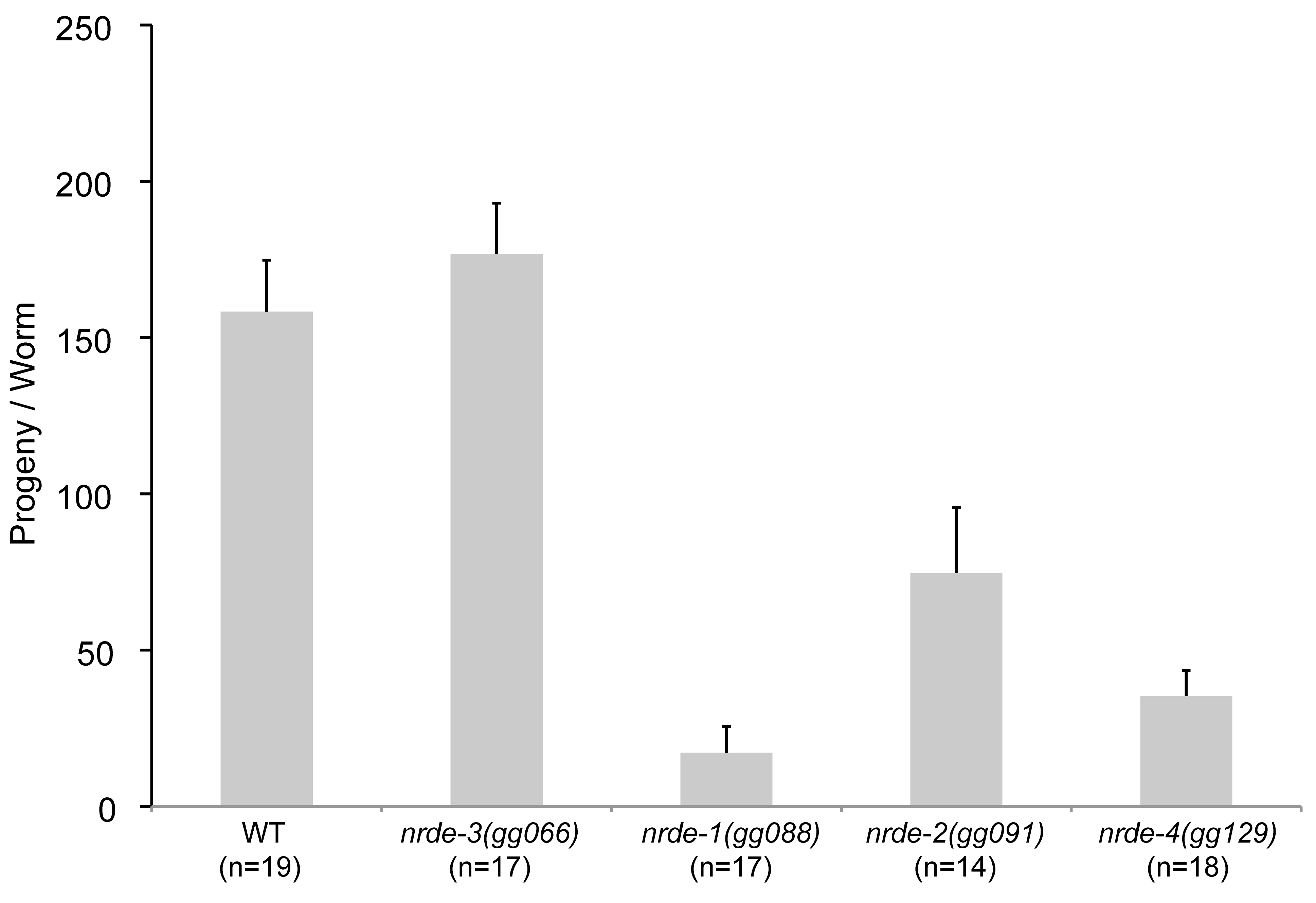

Supplement: Figure S9 — A subset of nrde mutants have reduced fecundity. nrde-1/-2/-4 mutants have reduced brood sizes. Brood sizes were counted from individual animals grown at 25°C. (error bars +/− s.d.). (TIF) [file pgen.1002249.s009.tif]
